# Dose-Dense Docetaxel versus Weekly Paclitaxel following Dose-Dense Epirubicin and Cyclophosphamide as Adjuvant Chemotherapy in Node-Positive Breast Cancer Patients: A Retrospective Cohort Analysis

**DOI:** 10.1155/2021/6653265

**Published:** 2021-09-20

**Authors:** Sara Khoshroo, Saleh Sandoughdaran, Parisa Sabetrasekh, Parastoo Hajian, Pegah Bikdeli, Parto Sabetrasekh, Fatemeh Nasrollahi, Ladan Mohammadi Yeganeh, Sepideh Jafari Naeini, Hamid Reza Mirzaei

**Affiliations:** ^1^Department of Radiation Oncology, Cancer Research Center, Shohadaye-Tajrish Hospital, Shahid Beheshti University of Medical Sciences, Tehran, Iran; ^2^Department of Neurological Surgery, The George Washington University School of Medicine and Health Sciences, Washington, DC, USA; ^3^Radiology Department, Advanced Diagnostics and Interventional Radiology Research Center (ADIR), Imam Khomeini Hospital, Tehran, Iran

## Abstract

**Methods:**

This study included patients from two prospective studies conducted in our institute from April 2007 to March 2009. Ninety-one women with axillary lymph node-positive breast cancer who had received four cycles of dose-dense epirubicin and cyclophosphamide were treated with either weekly paclitaxel (80 mg/m^2^) for 12 doses or biweekly docetaxel (75 mg/m^2^) for four cycles.

**Results:**

After a median follow-up of 88 and 109 months, 11 (23.4%) and 10 (22.7%) patients had experienced disease recurrence (*p* = 0.16), while 10 (21.3%) and 5 (11.4%) patients had died in the paclitaxel and docetaxel arm, respectively (*p* = 0.56). No significant difference could be seen in 5-year DFS or OS among groups (HR: 0.58; 95% CI: 0.19–1.81, *p* = 0.35; HR: 0.58; 95% CI: 0.19–1.81, *p* = 0.35, respectively).

**Conclusion:**

In conclusion, both evaluated adjuvant chemotherapy regimens have comparable effectiveness regarding DFS and OS.

## 1. Introduction

Breast cancer is the most common cancer in women worldwide, and it has been estimated that more than 35 percent of these patients present with axillary node metastasis [1, 2]. Although many advancements have been made in early diagnosis and treatment, breast cancer remains the leading cause of cancer-related mortality among women, taking the lives of over 450,000 annually worldwide [3].

Although surgery is still the primary treatment of choice, chemotherapy represents an integral component of adjuvant treatment in patients with breast cancer. At present, standard clinical practice in node-positive breast cancer patients is to administer a regimen of anthracycline-based chemotherapy followed by a taxane, either docetaxel or paclitaxel [4]. The results of a meta-analysis of 13 studies on over 23,000 women with high-risk, early-stage breast cancer indicated a significant enhancement in overall survival (OS) as well as disease-free survival (DFS) regardless of ER expression, nodal involvement, type of taxane, patient's age and menopausal status, and treatment schedule after adding taxanes to the anthracycline-based regimen [5].

Over the past few years, various attempts have been made to enhance the effectiveness of current chemotherapy protocols, including the introduction of dose-dense chemotherapy regimens. Dose-density chemotherapy refers to the administration of standard-dose chemotherapy with shorter intervals between the cycles, which has become a mainstay protocol for high-risk breast cancer patients [6]. Based on mathematical modeling of tumor growth, when the same doses of medications (which were used once in three weeks) are administrated once in two weeks, the rapid growth stage of the tumor cells will be interrupted [7]. Indeed, a meta-analysis assessing several randomized clinical trials (RCTs), in which dose-dense versus standard-schedule chemotherapy were compared in breast cancer, reported a significant survival benefit for dose-dense schedules in resected (mainly node-positive) disease [8].

Traditionally, docetaxel and paclitaxel have been considered to be similarly effective as adjuvant treatment in breast cancer. However, a phase 3 study by Sparano et al. reported that weekly paclitaxel is superior to weekly and triweekly docetaxel schedules in terms of both OS and DFS [9]. To the best of our knowledge, no study has compared weekly paclitaxel with a dose-dense regimen of docetaxel. The aim of the current study was to compare the outcome of node-positive breast cancer patients who had received weekly paclitaxel with those who were treated with dose-dense docetaxel postoperatively.

## 2. Methods

### 2.1. Patients

This retrospective study included patients from two prospective studies conducted from April 2007 to March 2009 at our institute [10, 11]. All patients were female with the inclusion criteria as follows: aged 18-80 years, had undergone primary surgery (mastectomy/lumpectomy), with a histologically confirmed invasive breast cancer and at least one histologically resected positive axillary lymph node; ECOG PS = 0, 1, adequate biological functions (hemoglobin of more than ten g/dl; absolute neutrophil count > 1.5 × 10^9^/l, platelet count > 100 × 10^9^/l, serum creatinine clearance of over 60 ml/min, bilirubin level below the upper normal limit (UNL), alkaline phosphatase (ALP) of less than 5 × UNL, aminotransferases of lower than 2.5 × UNL, and confirmed normal cardiac function (LVEF > 50%)). Patients with T4 stage or inflammatory breast cancer and those with prior anticancer therapy or other significant medical conditions were excluded.

All women received upfront epirubicin (100 mg/m2) and cyclophosphamide (600 mg/m^2^) every two weeks for four cycles. Women in the paclitaxel group were treated with 80 mg/m^2^ of paclitaxel given by iv infusion over 90 min weekly for 12 doses, and those in the docetaxel group received 75 mg/m^2^ of docetaxel given by iv infusion for one h every two weeks for four doses. All patients in the docetaxel group received granulocyte-colony-stimulating factor (G-CSF) 300 *μg* daily on days 3–10 of each course. Patients with estrogen- or progesterone-positive tumors received postoperative hormone therapy for five years or until disease recurrence.

### 2.2. Statistical Analysis

Overall survival and disease-free survival were estimated using the Kaplan-Meier method and Cox proportional hazards regression. Statistical analysis was carried out using IBM SPSS 22.0. All statistical tests were two-sided, and *p* < 0.05 was considered to be statistically significant.

## 3. Results

### 3.1. Patient Characteristics

From April 2007 till March 2009, 44 patients were treated with the dose-dense docetaxel, and 47 patients received weekly paclitaxel. As shown in Table 1, both groups were well balanced regarding baseline characteristics, except for age, as patients in the docetaxel group were significantly younger (*p* = 0.02). The median age was 42.5 years (range, 26–69 years) in the docetaxel group and 49 years (range, 31–71 years) in the paclitaxel group. Overall, the number of positive lymph nodes was 1–3 in 47.3% of patients, and involvement of more than ten nodes was observed in 17% of them. Estrogen and/or progesterone receptor-positive tumors were seen in about 70% of patients, while 30% were ER/PR negative.

### 3.2. Disease-Free Survival and Overall Survival

**Figure 1:**
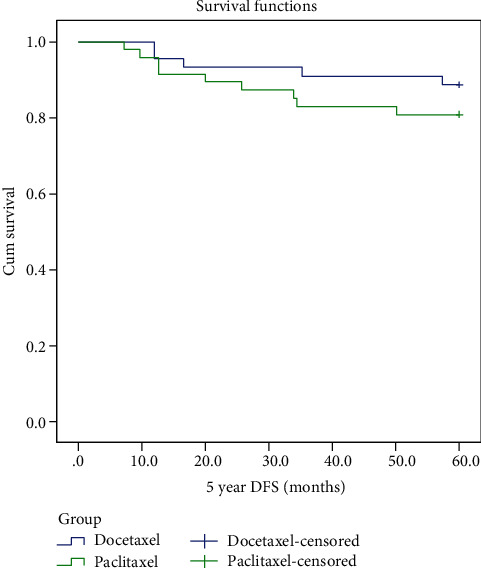
5-year disease-free survival in months for paclitaxel and dose-dense docetaxel as adjuvant chemotherapy for node positive breast cancer patients.

**Figure 2:**
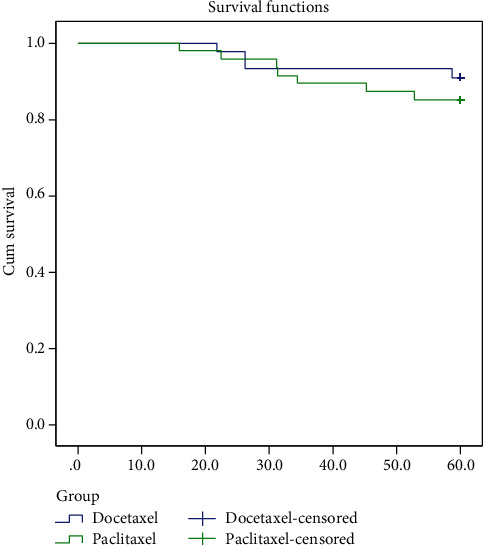
5-year overall survival in months for paclitaxel and dose-dense docetaxel as adjuvant chemotherapy for node positive breast cancer patients.

In order to compare the regimen efficacy, the DFS and OS of two groups were compared. At a median follow-up of 88 and 109 months, 10 (21.3%) and 5 (11.4%) patients had died (*p* = 0.56) and 11 (23.4%) and 10 (22.7%) patients had experienced disease recurrence (*p* = 0.16), in the paclitaxel and docetaxel arm, respectively (*p* = 0.56). Although the median DFS had not been reached yet, there was no significant difference in 5-year DFS between the paclitaxel- and docetaxel-treated group (HR 0.58; 95% CI 0.19–1.81; *p* = 0.35).  Figure 1 illustrates the Kaplan-Meier curves for 5-year DFS in the two treatment groups. The 5-year overall survival rate was 80.9% for the paclitaxel group and 88.6% for the group treated with the docetaxel group. Likewise, the median OS had not been reached in both groups and did not differ significantly between the two groups (HR 0.58; 95% CI 0.19–1.81; *p* = 0.35, respectively; Figure 2). Due to different baseline characteristics of patients in two groups, Cox regression analysis was performed, and results were confirmed on adjusted Cox regression proportional hazards analysis.

## 4. Discussion

The combined anthracycline and taxane therapy has been reported as a standard approach for the treatment of node-positive as well as high-risk node-negative early breast cancer patients [12]. While several studies have compared different taxane types and their administration schedules, no conclusion has been made regarding the optimum combination. In the largest trial, using a two × two factorial design, Sparano et al. randomized 4,954 node-positive breast cancer patients treated with four courses of doxorubicin and cyclophosphamide to receive either docetaxel or paclitaxel weekly for 12 weeks or every three weeks for four cycles [9]. Their report showed that although no differences were found regarding the comparisons of taxane type, paclitaxel 175 mg/m^2^ given every three weeks was associated with significantly lower DFS compared with weekly paclitaxel and three weekly docetaxel (HR, 0.84; *p* = 0.011 and HR, 0.79; *p* = 0.001, respectively).

Dose-dense chemotherapy, in which the dose intensity of the regimen is increased by delivering standard-dose chemotherapy with shorter intervals, is a widely accepted regimen for high-risk breast cancer patients. Recent trials have shown that dose-dense chemotherapy regimens increase both OS and DFS and are as safe as conventional 3-week interval regimens [13]. We have previously shown that dose-dense docetaxel is a feasible chemotherapy regimen [11]. However, it is not clear whether patients receiving dose-dense docetaxel have a better outcome than those receiving standard weekly paclitaxel regimen. In the current study, after a median follow-up of approximately seven years, no significant difference in DFS or OS was found between the patients receiving biweekly docetaxel compared to those treated with weekly paclitaxel. This finding is similar to those reported by Saloustros et al. who compared the efficacy of dose-dense docetaxel compared to dose-dense paclitaxel in node-positive early breast cancer patients [14]. In their research, following surgery node-positive HER2-negative breast cancer patients were randomized to receive four courses of either biweekly docetaxel (75 mg/m^2^) or paclitaxel (175 mg/m^2^) following four cycles of 5-fluorouracil, epirubicin, and cyclophosphamide (700/75/700 mg/m^2^). In concordance with the results of our study, after a median follow-up of 6 years, they reported no significant difference in DFS or OS between the paclitaxel and docetaxel-treated patients.

This study has some limitations which have to be pointed out. First, this was a retrospective study, and patients were not randomized. Second, data on grading and lymphovascular invasion and perineural invasion were not available for a number of cases, which could affect treatment results and make our conclusions less definitive. In order to tackle these limitations, a prospective study with more cases is required.

In conclusion, both evaluated adjuvant chemotherapy regimens have comparable effectiveness regarding DFS and OS. Due to the higher rate of toxicity reported in patients receiving dose-dense docetaxel in previous studies, paclitaxel remains the taxane of choice for treating node-positive breast cancer patients.

## Figures and Tables

**Table 1:** Patient characteristics.

Characteristic	Dose dense docetaxel group (*n* = 44)	Weekly paclitaxel group (*n* = 47)	*p* value
Mean age (yrs)			
Standard deviation	45.19 ± 9.8	55.13 ± 10.4	0.02
Range	26-69	31-71	
Pathology			
IDC	42 (95.5)	46 (97.9)	0.335
Other	2 (4.5)	1 (2.1)	
T stage			
T_1_ -T_2_	28 (68.3)	35 (81.4)	0.16
T_3_ -T_4_	13 (31.7)	8 (18.6)	
N stage			
N_1_	19 (46.3)	24 (53.3)	0.731
N_2_	14 (34.1)	12 (26.7)	
N_3_	8 (19.5)	9 (20)	
Hormone receptors			
ER(+)/PR(+)	26 (59.1)	27 (57.4)	0.59
ER(+)/PR(-)	3 (6.8)	7 (14.9)	
ER(-)/PR(+)	2 (4.5)	1 (2.1)	
ER(-)/PR(-)	13 (29.6)	12 (25.6)	
HER2 status			
Positive	32 (72.7)	28 (71.8)	0.92
Negative	12 (27.3)	11 (28.2)	

Data presented as no. (%) unless otherwise specified.

## Data Availability

The research data used to support the findings of this study are restricted by the Shohaday-e-Tajrish Hospital ethics board in order to protect patient privacy.
